# Is central vision (and a fovea) needed for emmetropisation?

**DOI:** 10.1111/opo.13494

**Published:** 2025-03-20

**Authors:** Frank Schaeffel, Christine Wildsoet, Ranjay Chakraborty

**Affiliations:** ^1^ Section of Neurobiology of the Eye Institute for Ophthalmic Research, University of Tuebingen Tuebingen Germany; ^2^ Institute for Molecular and Clinical Ophthalmology Basel (IOB) Basel Switzerland; ^3^ Zeiss Vision Science Lab Institute for Ophthalmic Research, University of Tuebingen Tuebingen Germany; ^4^ School of Optometry University of California, Berkeley Berkeley California USA; ^5^ Myopia and Visual Development Lab Caring Futures Institute, College of Nursing and Health Sciences, Sturt North, Flinders University Adelaide South Australia Australia

## INTRODUCTION

**Ranjay Chakraborty**

Investigations into the neural circuits underlying emmetropisation have identified key components responsible for converting visual input into molecular signals that regulate eye growth. Studies in chicks and primates have shown that isolating the eye from higher brain centres through surgical transection of the optic nerve does not prevent form‐deprivation myopia or lens‐induced myopia in chicks. In addition, eliminating the autonomic neural input to the eye's accommodative system does not affect the visual regulation of ocular growth in chicks. Furthermore, in both chicks and primates, partial diffusers applied to only half of the visual field resulted in localised ocular elongation and myopia in the corresponding half of the eye. Research conducted across multiple laboratories and species has consistently demonstrated that the eye' ability to respond to optical manipulations is linked to a local, regionally selective retinal mechanism within the eye.

The local nature of the vision‐dependent emmetropisation mechanisms has significant implications for refractive development, particularly in primates with a foveal retina specialised for central vision. Although the fovea, with its high cone photoreceptor density, has traditionally been assumed to dominate axial growth and refractive development, emerging evidence challenges this assumption. The refractive state at the fovea is influenced by changes in both the posterior pole and the periphery of the eye (e.g., tangential scleral expansion in the periphery can promote central axial elongation), indicating that peripheral visual signals could independently affect eye shape and central refractive development, depending on how retinal mechanisms alter peripheral scleral changes. This point–counterpoint article evaluates the evidence on whether central vision from the fovea is essential for emmetropisation and discusses the features of local growth‐regulating mechanisms in the retina.

## POINT

**Frank Schaeffel**

Humans have a high visual acuity fovea, but that is not the case for all non‐primate mammalians. Is the human fovea important for refractive development, or is it a case of the human using the baseline mammalian system, with the fovea ‘along for the ride’? We hypothesise that emmetropisation is an old and conserved process, and consider the evolutionary history of vertebrate visual systems.

Chickens have no fovea but rather an ‘area centralis’ with higher cone density than in the periphery and almost no rods.^1^, ^2^ The area centralis in chicks has a diameter of about 3 mm, which is equivalent to about 27° in the visual field (calculated from a retinal image magnification of 116 μm/° in a 30‐day‐old chicken with an axial length of 10.54 mm).^3^ Therefore, it is much larger than typically covered by a fovea. Nevertheless, the chicken became an important model in myopia research and has provided a number of important discoveries, such as that deprivation myopia develops locally when only parts of the visual field were covered by a diffuser^4^ or that growth changes can be induced in only one half of the eye when defocus was imposed by hemifield lenses.^5^ Furthermore, if only the periphery of the visual field was defocused by a lens that had a hole in its centre, then just the peripheral area compensated for the power of the lens while the refractive state remained unchanged in the centre.^6^ Equally striking was that chickens, kept under low ceilings in their cages, developed myopia in their upper visual field only.^7^ All this took place with intact accommodation, which shifted the plane of focus coherently over the entire visual field when accommodation was engaged (chickens,^8^ humans^9^). However, are all of these advanced capabilities a special feature of the chicken eye? It seems unlikely, because other animal models of myopia also emmetropise successfully without a fovea, such as guinea pigs with a visual streak,^2^, ^10^ mice with a visual streak^2^ and tree shrews with an area centralis,^2^ a region of high ganglion cell density at the posterior part of the eye that looks forwards.^11^ However, unlike primates, there is no higher density of cones over this pseudo‐fovea^12^ but rather a modest superior to inferior retinal gradient in cone density. Overall, all have retinal areas with a higher photoreceptor and ganglion cell density, but no retinal location where the inner retinal layers are pushed aside, forming a narrow pit that defines the presence of a fovea and covers only a small fraction of the visual field. In human eyes, the fovea has a diameter of about 0.65–0.7 mm,^13^ equivalent to about 2° in the visual field, but it covers less than 1% of the total retinal area. There is evidence that the fovea is not needed for emmetropisation in monkeys: When this highly specialised retinal area was ablated by laser photocoagulation, these monkeys displayed normal emmetropisation and developed deprivation myopia with diffusers, similar to untreated controls.^14^ Furthermore, foveal ablation did not change the pattern of peripheral refraction, neither when they had normal vision nor during the development of deprivation myopia.^15^

In the course of evolution, a fovea first appeared in the temporal retina of predatory fish.^16^ It is unclear whether amphibia ever developed a fovea, but salamanders do not have one.^17^ A fovea is found in a number of reptiles like chameleons,^18^ anolis^19^ and geckos.^20^ A fovea is also present in birds of prey, such as falcons^21^, ^22^ and eagles.^23^ Even pigeons have a fovea.^24^ There is no convincing literature as to whether dinosaurs, common ancestors of birds and mammals had already developed a fovea. However, it is clear that a fovea later disappeared in mammals, perhaps related to their initially nocturnal and underground living habits.^16^ Strikingly, a fovea is still lacking in all mammals, except for the anthropoid lineage (monkeys and humans), where it was apparently ‘reinvented’ over the last 35–40 million years.

One could argue that emmetropisation is only necessary when visual acuity is high, or expressed the other way around, visual acuity can only be high with a fovea. However, many mammals have good visual acuity without a fovea, such as horses (23 cycles per degree^25^) or dogs (9.5 cycles per degree in the beagle^26^), both of whom only have a visual streak. This suggests that visual control of eye growth was successful without a fovea. Further, if only the fovea would emmetropise the eye, then it would be difficult to explain how the retinal plane can be matched to the image shell all over the visual field (e.g., in chickens^8^ or humans^27^). Pigeons, who have a fovea, develop ‘lower field myopia’,^28^ and traces of ‘lower field myopia’ can even be found in humans,^27^, ^29^ both indicators of peripheral emmetropisation.

In the periphery, human visual acuity drops to one‐tenth of that at the fovea, but there is still emmetropisation. Refractive errors are very low within the central ±15°.^27^ However, if the refractive error is roughly constant across the surface of the retina—at least within the region likely important for human refractive development—then it stands to reason that optimising focus at the near/mid periphery would also optimise focus at the fovea. That is, there would be no reason for emmetropisation to target the fovea specifically. The lower visual acuity in the periphery is not a problem since recent experiments have confirmed that high visual acuity is not needed for emmetropisation^30^ or accommodation.^31^ It was found that spatial frequencies <8 cycles per degree are sufficient to elicit bidirectional short‐term changes in axial length in young human subjects, when defocus was imposed.^30^ Finally, if the fovea was gaze‐contingently patched by means of eye tracking while subjects watched a movie, the eyes still responded to positive lenses as with an unobstructed fovea, with axial shortening.^32^ Interestingly, the eyes no longer responded when an annular area of 6–9° eccentricity around the fovea was gaze‐contingently patched with a grey field. Apparently, the perifoveal area is crucial for emmetropisation in humans, perhaps because S‐cones are abundant and can provide information on longitudinal chromatic aberration, a powerful cue to derive the sign of defocus. The role of the rods, which cover >90% of the peripheral retina, remains unclear, but it seems likely that they are involved, in particular since their absorption spectrum overlaps widely with the S‐cones. In summary, there is currently no clear evidence that the fovea is needed for emmetropisation in humans and other foveate primates.

## COUNTERPOINT

**Christine Wildsoet**

The debate over the contributions of the central as opposed to the peripheral retina to eye growth regulation, and so the development of myopia, has been both fierce and extended. Today, there is very little dispute over the existence of an active, optical defocus‐driven emmetropisation process that serves to fine‐tune early eye growth to eliminate neonatal refractive errors, and furthermore, that the controller resides within the eye. Much like ocular accommodation, this ocular growth controller serves to correct for optical defocus, albeit more enduringly. As myopia prevalence figures climb to reach epidemic levels in many parts of the world,^33^ interest has grown rapidly in how one might manipulate the output of this controller with appropriately designed optical devices to prevent or slow myopia progression. To this end, a key related question is what region or regions of the retina are responsible for decoding the sign of imposed optical defocus. The evidence for a primary role for the central retina is overwhelming, as summarised below.

### Retinal evidence

While there are promising data from animal studies hinting at important contributions from more peripheral retinal regions in eye growth regulation, one must also consider species differences in retinal anatomy and function before extrapolating to the human eye. Of relevance to this debate is that in the case of the human retina, cones are largely confined to the central 10°, reaching a peak in the foveola (160,000 cones/mm^2^), and decreasing to 5000 cones/mm^2^ at around ±20° eccentricity, with the fraction of the retinal image captured by the cone mosaic (i.e., coverage factor), being relatively stable beyond 15°.^34^ Of relevance to this debate, and arguing for a key role of the central retina, is the observed central bias in early changes of retinal function detected with multifocal (MF) electroretinograms (induced component) in children exhibiting early myopic shifts in refractive error.^35^ Furthermore, the most commonly cited evidence against a key role for the central retina in eye growth regulation, namely, that from Smith's laboratory,^14^, ^15^, ^36^ is not as robust as it might seem. This group reported recovery from induced myopia in infant monkeys after removal of the form depriving diffusers, even when the central 5°–6° of retina was first rendered non‐functional by laser ablation.^36^ However, the data for animals subjected to the laser treatment show significant within‐ and between‐animal variability in changes in refractive error over time.^14^, ^15^ Also not considered in interpreting the results from these studies is the high probability that the laser surgery triggered local changes in the expression of multiple growth factors, with one or more having potential confounding effects on ocular growth. Indeed, in a study on mice, argon laser photocoagulation significantly altered the expression of 265 genes, tied to a range of biological structures and/or processes, with FGF‐14 and FGF‐16 among those downregulated.^37^ The possible contribution of such nonspecific, inhibitory effects on ocular growth to the reported recovery responses cannot be excluded, given that the studies in monkeys did not include any gene or protein analyses and only limited biometric (vitreous chamber depth) data are reported. Finally, given the young ages of the animals and relatively short monitoring periods (animals <6 months of age at the end of the monitoring period, except for one case), nonvisual, developmental drivers of ocular growth represent yet another confounding factor that cannot be easily disentangled.^38^

### Scleral evidence

As the outermost layer of the posterior vitreous chamber wall, the sclera serves to provide protection to the inner structures, including the neural retina. In mammalian and primate eyes, the sclera is composed of a dense network of collagen fibres, embedded in extracellular matrix (ECM), and maintained by a sparse population of interspersed scleral fibroblasts, which are both the source of collagen and responsible for ongoing ECM remodelling. The latter is upregulated in myopia progression, as manifested in accelerated vitreous chamber enlargement, with regional variation in the same reflected in differences in the scleral thickness profiles of emmetropic and myopic eyes (see review^39^). Specifically, for eyes that remain emmetropic, the sclera tends to be thickest at the posterior pole, that is, underlying the central retina, with the equatorial regions being relatively thinner.^40^, ^41^ In contrast, the sclera at the posterior pole of eyes exhibiting myopia shows relative thinning, becoming more exaggerated with increasing eye length,^40^, ^41^, ^42^ and consistent with local ‘myopia‐genic signals’ being generated by the fovea and relayed via the retinal pigment epithelium to the nearby sclera.^43^ In parallel, eye shape, which is largely a by‐product of local biomechanical interactions between the sclera and intraocular pressure,^40^ changes. Specifically, eyes become increasingly prolate with increased scleral thinning at the posterior pole, which is also consistent with the more hyperopic relative peripheral errors recorded in these faster ‘growing’ eyes.^44^ Such shape changes, also observed experimentally in young monkeys fitted with full‐field negative lenses, exaggerate rather than correct the peripheral retinal defocus experience.^15^ It is also arguable that the changes in peripheral refractive errors observed immediately before, as well as after, the onset of myopia in children^44^ are a by‐product of enhanced scleral remodelling at the posterior pole, given that these eyes also show accelerated axial elongation before exhibiting myopic refractive errors. The assumed trigger is myopia‐genic foveal stimulation.

### Emmetropisation as an ocular focussing mechanism

While the relative importance of foveal versus para‐ and perifoveal input to accommodation accuracy has long been a topic of interest among those studying accommodation, the potential significance of the answer to this question has grown in the search for optical interventions for controlling myopia. In this context, a recent study by Sharmin et al.,^45^ making use of a tunable lens to compare the parafoveal and perifoveal accommodation responses to defocus changes, is highly relevant. Here, young adult subjects exhibited increases in response times and decreases in response amplitudes with increasing eccentricity, becoming negligible at a 7° radial distance. In other words, foveal input was synonymous with optimal accommodation, with no differences in performance tied to the refractive error observed in this case. If emmetropisation can be viewed as just another ocular focusing mechanism, like accommodation, then one would expect foveal input to dominate.

### Myopia control and MF optical interventions

Of studies making use of novel spectacle lens designs in animal models of myopia, one of the first involved young chicks and two‐zone spectacle lenses undertaken in my research laboratory.^46^ When either myopic (+) or hyperopic (−) optical defocus was limited to either a central or peripheral lens zone (with the remaining area being afocal), similar growth response patterns to those seen with single vision lenses were recorded, with only the magnitude of the response varying with the size of the optical defocus zone. Notably, a closer look at the data for the two‐zone centre plano/peripheral +5 D lenses reveals responses in excess of those with a single vision +5 D lens for all but the lens design with the narrowest peripheral +5 D zone. That the retinal defocus experience was modified through interactions between the spherical aberration inherent in the lenses and the negative spherical aberration inherent in the chick eye was considered among plausible explanations for this apparent discrepant result^47^ and one with precedence. In a study comparing monocular accommodation responses in human subjects wearing MF soft contact lenses incorporating a +1.5 D peripheral add,^48^ leads of accommodation (as derived from consensual responses) were observed, opposite to that predicted based on the assumed reduced accommodative demand experienced by the peripheral retina, but consistent with the predicted foveal defocus experience, based on results from optical modelling of interactions between the eye's own aberrations and those of the lenses.^49^ These accommodation data also predict slowed progression in MF soft contact lens wearers, as reported in an early study using MF contact lenses off‐label^50^ and in more recent studies using custom‐designed myopia control contact lenses.^51^ Might the same explanation hold for the myopia control effects of orthokeratology lenses, for which induced changes in spherical aberration yield ocular optical profiles similar to those achieved with centre distance MF soft contact lenses? Together, the results from these various studies argue for a key role of the fovea in eye growth regulation (and dysregulation). Results from two additional, unrelated studies involving MF electroretinogram recordings in the presence of dual focus and extended depth of focus contact lenses^52^, ^53^ are also consistent with significant central retinal contributions to their myopia control actions.

The discussion on MF optical devices would not be complete without mention of the emerging array of novel myopia control spectacle lens designs.^54^ While a key role for the peripheral retina in eye growth regulation is the underlying premise for their designs, which all limit ‘treatments’ to more peripheral lens areas, it must also be acknowledged that human eyes are in constant motion, thereby exposing the fovea to the modified peripheral lens zones, with less or more frequency as the environment is scanned. Given that all but one design involved positive power lenslets, an early study in the chick from the Wallman laboratory^55^ is of potential relevance. Here just six, 2‐min exposures to myopic (+) defocus per day were sufficient to slow eye elongation, while the response to imposed hyperopic defocus (the equivalent of accommodative lags in humans) was abolished by just 8 min of daily positive lens wear. Could the diffusers used in place of defocusing lenslets, as in diffusion optics technology lenses (sightglassvision.com), similarly disrupt the myopia‐genic effect of extended near work and exposure to lags of accommodation? Data from two other studies from the same group^56^, ^57^ hint at this possibility.

In summary, from an evolutionary design perspective, the structure and function of the human eye are logical, based on a need to obtain through development, and thereafter to maintain, optimal focus of key features of interest in the visual environment. To this end, a central retinal area (fovea and near surround) with high spatial resolution capacity and an ability to decode and correct for enduring suboptimal defocus are essential prerequisites.

## SUMMARY

**Ranjay Chakraborty**

### Points of agreement

- The primary vision‐dependent mechanisms regulating eye growth and refractive development are located within the retina and operate in a region‐specific manner.
- In humans, the highest cone density in the fovea corresponds to the best visual acuity. However, certain visual functions that are potentially important for regulating emmetropisation, such as short‐term axial length changes in response to defocus, the accommodative response or grating detection, do not rely on high‐quality vision, suggesting a role of the peripheral retina in eye growth modulation.
- There are significant anatomical and functional differences in the fovea and retinal system across species (e.g., the presence of a fovea in primates vs. an area centralis in chickens and a visual streak in mice and guinea pigs), which may influence how the fovea processes vision and regulates ocular growth.

### Issues to be resolved

- Do neurons in the peripheral retina, with larger receptive fields and lower spatial resolution, actively decode defocus signals? How are these signals transmitted to the choroid and retinal pigment epithelium to regulate ocular growth?
- If ocular growth is primarily driven by signals from the peripheral retina, why does the sclera at the posterior pole, underlying the central retina, become progressively thinner in myopia, eventually predisposing the eye to staphyloma and myopic maculopathy, compared to the peripheral or equatorial regions of the sclera? Does the thinning and remodelling of the sclera at the posterior pole trigger further changes in the peripheral regions of the sclera during pathological myopia?
- If the para‐ and perifoveal retina are critical for eye growth, can we identify focal regions in the peripheral retina and choroid that respond strongly to defocus or visual environmental manipulations, perhaps using wide‐field optical coherence tomography imaging and multifocal electroretinogram?
- Could the myopia control effects observed with novel multifocal spectacle lens designs be partially explained by intermittent foveal exposure to peripheral lens zones during natural eye movements rather than solely by peripheral retinal signalling?
- The visual system may process signals from both the foveal and peripheral regions, with the direction and nature of eye growth dictated by the spatial summation of visual signals across the entire retina, rather than from one region alone. Are there specific areas in the retina that play a dominant role in the spatial integration of these signals, and do these areas respond differently to hyperopic versus myopic defocus? For example, both peripheral form deprivation^36^ and peripheral hyperopic defocus^58^ led to central axial myopia in monkeys, with the range and average myopic refractive errors being comparable to those induced by full‐field treatment lenses. In contrast, peripheral myopic defocus slowed axial elongation, resulting in central hyperopia, with the degree of hyperopia being greater than that produced by full‐field positive lenses in chicks.^59^

## CONFLICT OF INTEREST STATEMENT

The authors declare no conflicts of interest.
